# Common and uncommon neurological manifestations of neuroborreliosis leading to hospitalization

**DOI:** 10.1186/s12879-016-2112-z

**Published:** 2017-01-21

**Authors:** Philipp Schwenkenbecher, Refik Pul, Ulrich Wurster, Josef Conzen, Kaweh Pars, Hans Hartmann, Kurt-Wolfram Sühs, Ludwig Sedlacek, Martin Stangel, Corinna Trebst, Thomas Skripuletz

**Affiliations:** 1 Department of Neurology, Hannover Medical School, Carl-Neuberg-Str. 1, 30625 Hannover, Germany; 2 Department of Diagnostic and Interventional Neuroradiology, Hannover Medical School, Hannover, Germany; 3 Department of Paediatrics, Hannover Medical School, Hannover, Germany; 4 Institute for Medical Microbiology and Hospital Epidemiology, Hannover Medical School, Hannover, Germany

## Abstract

**Background:**

Neuroborreliosis represents a relevant infectious disease and can cause a variety of neurological manifestations. Different stages and syndromes are described and atypical symptoms can result in diagnostic delay or misdiagnosis. The aim of this retrospective study was to define the pivotal neurological deficits in patients with neuroborreliosis that were the reason for admission in a hospital.

**Methods:**

We retrospectively evaluated data of patients with neuroborreliosis. Only patients who fulfilled the diagnostic criteria of an intrathecal antibody production against Borrelia burgdorferi were included in the study.

**Results:**

Sixty-eight patients were identified with neuroborreliosis. Cranial nerve palsy was the most frequent deficit (50%) which caused admission to a hospital followed by painful radiculitis (25%), encephalitis (12%), myelitis (7%), and meningitis/headache (6%). In patients with a combination of deficits, back pain was the first symptom, followed by headache, and finally by cranial nerve palsy. Indeed, signs of meningitis were often found in patients with neuroborreliosis, but usually did not cause admission to a hospital. Unusual cases included patients with sudden onset paresis that were initially misdiagnosed as stroke and one patient with acute delirium. Cerebrospinal fluid (CSF) analysis revealed typical changes including elevated CSF cell count in all but one patient, a blood-CSF barrier dysfunction (87%), CSF oligoclonal bands (90%), and quantitative intrathecal synthesis of immunoglobulins (IgM in 74%, IgG in 47%, and IgA in 32% patients). Importantly, 6% of patients did not show Borrelia specific antibodies in the blood.

**Conclusion:**

In conclusion, the majority of patients presented with typical neurological deficits. However, unusual cases such as acute delirium indicate that neuroborreliosis has to be considered in a wide spectrum of neurological diseases. CSF analysis is essential for a reliable diagnosis of neuroborreliosis.

## Background

Lyme Borreliosis is a tick-borne transmitted infectious disease caused by the spirochete Borrelia burgdorferi sensu lato. This spirochete can invade the central nervous systems (CNS) resulting in neuroborreliosis in up to 15% of the affected patients [1, 2]. The clinical course of neuroborreliosis is highly variable [3, 4]. Meningoradiculitis, also described as Bannwarth’s syndrome, is the most frequent manifestation of neuroborreliosis in Europe [5]. Symptoms include headache, cranial nerve palsy, and/or lancinating pain. Although at least 80% of European patients present with facial nerve palsy and radiculitis, symptoms of neuroborreliosis may be quite unspecific or even mimic other neurological diseases [3, 6]. Myelitis and encephalitis are rare clinical manifestations [6]. To date, different stages and syndromes of neuroborreliosis have been described with up to ten subgroups [4].

The onset of neuroborreliosis is usually subacute with progression over weeks. However, cases of acute stroke-like symptoms and chronic encephalitis have also been described [2, 7]. In clinical practice, patients are usually categorized into acute neuroborreliosis (symptom duration < 6 months) and late manifestation/chronic neuroborreliosis (symptom duration > 6 months) [4, 8].

The diagnosis of neuroborreliosis is based on medical history, clinical findings, serological and cerebrospinal fluid analysis (CSF) [6, 9]. Detection of pleocytosis, blood-CSF-barrier dysfunction, intrathecal production of immunoglobulins (Ig) and especially an intrathecal synthesis of Borrelia specific antibodies in CSF are the best indicators for definitive diagnosis [10]. Once diagnosed, the majority of patients with neuroborreliosis experience a favorable outcome after antibiotic treatment [11–15]. However, in a small number of patients residual symptoms remain [16].

Here, we performed a thorough evaluation of clinical and laboratory data in patients with neuroborreliosis. The aim of this retrospective study was to define pivotal neurological deficits in patients with neuroborreliosis being the reason for admission to a hospital.

## Methods

### Patients

The retrospectively evaluated data originate from 68 patients. All data were collected for routine diagnostics at the Hannover Medical School in the time from 1999 to 2014. Only patients who fulfilled the diagnostic criteria of an intrathecal antibody production against Borrelia burgdorferi sensu lato were included in the study [8]. The investigation was approved by the institutional ethics committee.

### CSF and serum analytical procedures

CSF and serum were analysed by routine methods [17–19]. CSF cells were counted manually with a Fuchs-Rosenthal counting chamber. CSF total protein was determined by the Bradford dye-binding procedure. IgG, IgA, IgM, and albumin were measured in CSF and serum in the same latex enhanced assay by kinetic nephelometry (Beckman Coulter IMMAGE). Blood–CSF barrier function was assessed by CSF-serum albumin quotients (QAlb) [20]. Intrathecal synthesis of IgG, IgA, and IgM was calculated based on the method of Reiber-Felgenhauer referring the IgG, IgA, and IgM quotients to the albumin quotient [20]. CSF-specific oligoclonal bands (OCB) were determined by isoelectric focusing in polyacrylamide gels with consecutive silver staining.

IgM and IgG antibody production against Borrelia burgdorferi sensu lato was determined in serum and CSF by enzyme-linked immunosorbent assays (ELISA) according to the instructions of the manufacturer (*recom*Well Borrelia® Mikrogen). Western blots (ViraStripe® Viramed) were performed to confirm positive ELISA results. Intrathecal synthesis of Borrelia burgdorferi sensu lato specific IgG and IgM antibody specific index (AI) was calculated according to the formula (CSF Ig Borrelia/serum Ig Borrelia)/(CSF Ig total/serum Ig total) [21]. In case of intrathecal synthesis of immunoglobulins G and M the following formula was used: (CSF Ig Borrelia/serum Ig Borrelia)/Qlim. Qlim represents the Ig fraction in CSF originating only from blood and was calculated according to the individual’s albumin quotient [21]. AI values ≥ 1.5 indicate specific antibody synthesis in the CNS. The AI could be calculated in 62 patients. In 6 patients antibodies against Borrelia burgdorferi sensu lato were found significantly higher in CSF than in serum proving an intrathecal synthesis (five patients with IgM antibodies and one patient with IgG antibodies).

## Results

### Patient’s characteristics

In the period from 1999 to 2014 a total of 68 patients were hospitalized with neuroborreliosis. A male predominance was identified (65%; Table 1). The median age of all patients at presentation was 45 years. Our cohort included 11 children between ages 5 and 14 years. Immunosuppression as predisposing factor for infectious diseases was observed in three patients (melanoma, Crohn’s disease, polyarthritis). Our patients were categorized into five groups depending on the dominant neurological deficit that caused admission to our hospital (Table 1): cranial nerve palsy, symptoms/signs of radiculitis (back pain alone or combined with radiating pain into the limbs without or with additional limb palsy), symptoms/signs of meningitis (headache with or without neck stiffness), symptoms/signs of encephalitis, and symptoms/signs of myelitis.

**Table 1:** Patient’s characteristics. Age and duration of symptoms to diagnosis are presented by median with lowest and highest values

Clinical features	Patients (number)	Age (years)	Males (number)	Tick bite and/or erythema migrans (number)	Duration of symptoms (days)
All patients	68	45 (5–93)	44/68	23/68	16 (1–733)
Cranial nerve palsy	34	40 (6–77)	23/34	10/34	7 (1–121)
Facial nerve palsy	29	34 (6–77)	16/29	9/29	7 (1–121)
Isolated facial nerve palsy	11	26 (6–73)	7/11	4/11	4 (1–15)
+ Radiculitis	8	57 (18–77)	6/8	2/8	18 (2–49)
+ Meningitis	6	67 (30–93)	4/6	2/6	7 (3–21)
+ Radiculitis + meningitis	4	(34, 43, 44, 57)	3/4	1/4	(2, 21, 28, 121)
Oculomotor nerve palsy	5	53 (38–76)	2/5	1/5	53 (38–76)
Isolated oculomotor nerve palsy	2	(76, 42)	1/2	0/2	(2, 7)
+ Meningitis/Radiculitis	3	(26, 38, 53)	1/3	1/3	(11, 25, 26)
Radiculitis	17	67 (5–93)	10/17	9/17	20 (3–379)
Isolated radiculitis	14	67 (5–93)	8/14	8/14	18 (3–379)
+ Meningitis	3	(13, 65, 75)	2/3	0/3	(3, 22, 30)
Encephalitis	8	70 (8–79)	7/8	2/8	183 (1–732)
Chronic course	5	74 (67–79)	4/5	0/5	186 (81–732)
Acute/Subacute onset	3	(8, 11, 45)	3/3	2/3	(1, 1, 38)
Myelitis	5	37 (7–64)	4/5	0/5	126 (23–733)
Meningitis	4	(22, 34, 45, 64)	0/4	2/4	(1, 4, 7, 120)

### Patient’s signs and symptoms

Twenty-three patients (34%) reported previous tick bite and/or erythema chronicum migrans (Table 1). Nine of them remembered only tick bite, seven remembered the occurrence of erythema chronicum migrans with previous tick bite, while seven remembered erythema chronicum migrans without previous tick bite.

**Fig. 1:**
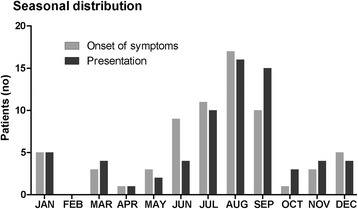
Seasonal distribution of patients with neuroborreliosis. Graph shows onset of symptoms and presentation in our hospital

In the majority (47 patients, 69%), onset of symptoms occurred between June and September (Fig. 1). 41 patients (60%) presented between July and September in our hospital. The median duration from onset of symptoms until diagnosis was 16 days (Table 1). A chronic course of the disease with symptoms persisting for longer than 6 months was found in 7 patients (10%). Five of them were diagnosed with chronic encephalitis, one presented with myelitis and one with radiculitis.

### Cranial nerve palsy

Cranial nerve palsy was the most frequent symptom that led to hospitalization (50%). In this group, 29 patients (43%) presented with facial nerve palsy and five patients (7%) with oculomotor disturbance due to abducens or oculomotor nerve palsy (Table 1).

Isolated facial nerve palsy was found in 11 patients (16%). Except three patients with bilateral facial palsy, clinical examination could not distinguish these patients from an idiopathic facial palsy (Bell’s palsy). The other patients with facial nerve palsy showed a combination with radiculitis (12%), meningitis/headache (9%), or both radiculitis and meningitis (6%). Patients with facial nerve palsy and radiculitis suffered from severe back pain radiating into the limbs and three patients even developed limb palsy. In all but one of them back pain preceded facial nerve palsy. In the last patient facial nerve palsy and limb palsy occurred suddenly.

All patients with facial nerve palsy and meningitis reported headache as the first symptom but facial nerve palsy was the principal reason for presentation to our hospital.

The combination of facial nerve palsy, meningitis, and radiculitis was found in four patients (6%). In all of these patients back pain was the first symptom, followed by headache, and finally by facial nerve palsy. One of them was initially misinterpreted as stroke.

Oculomotor disturbance occurred in five patients. One patient presented with isolated abducens nerve palsy and another patient showed isolated oculomotor nerve palsy. Two patients showed a combination of abducens nerve palsy and meningitis and one presented with abducens nerve palsy and signs of radiculitis. The latter three patients suffered from headache or back pain prior to abducens nerve palsy.

### Radiculitis

Symptoms of radiculitis without any affection of cranial nerves were found in 17 patients (25%). Ten patients presented with diffuse back pain and four of them reported radiating pain into the legs and two into the arms. Another seven patients sustained radiating back pain combined with unilateral weakness (four with leg palsy, two with arm palsy). In one patient arm and leg weakness developed suddenly, and thus, the patient was initially misinterpreted as stroke. During the disease course three patients with radiating back pain developed signs of meningitis.

### Encephalitis

Eight patients were classified as having encephalitis (12%). Three patients presented with symptom duration of less than 4 weeks and were thus classified as having acute encephalitis. One patient presented with clinical signs of delirium for hours. One 8-years old patient was admitted with four episodes of aphasia and dizziness with gait disturbance occurring on the day of presentation without any MRI abnormalities of the brain. One 11-years old patient reported headache over 3 weeks with ataxia and tremor in both arms and legs. MRI of the brain identified one T2-lesion in the left basal ganglia and another one in the left parietal lobe. EEG showed spike wave discharges over left temporo-occipital regions.

Five patients described a slow onset of symptoms with duration of more than 6 months and were thus classified as having chronic encephalitis. All these patients were older than 65 years and revealed cognitive impairment and gait disorder. MRI of the brain did not show any signs of inflammation but signs of vascular changes and brain atrophy.

### Myelitis

Five patients presented with signs of myelitis (7%). Paraspasticity was found in four patients of whom two suffered from weakness in both legs. One patient without spasticity showed weakness in both arms and reported transversal impairment of sensation (below T5). Bladder and rectum function were normal in all patients. Furthermore, these patients did not suffer from cranial nerve palsy or headache. MRI imaging of the spine confirmed a diagnosis of myelitis in all five patients. Lesions were located in the cervical spinal cord in one patient (C1), in the thoracic spinal cord in two patients (T1, T7-T12), and crossing the cervical and thoracic spinal cord in two patients (C7-T1, C3-T6). In addition, MRI showed an affection of dorsal root nerves in two patients.

### Meningitis

Four patients presented with headache only (6%). During clinical examination neck stiffness was found in three of them. Headache was described as diffuse with holocephal localization in three patients and with alternating localization in one patient. Concomitant fever was found in only one patient.

### Cerebrospinal fluid analyses and antibody response

CSF analysis was performed in all patients (Table 2). All but one patient showed elevated CSF cell counts. Total protein was increased in 58 patients (85%). The albumin CSF-serum concentration quotient (QAlb) is generally considered as the best indicator for a blood-CSF barrier dysfunction [20, 22]. Measurements of QAlb revealed age-corrected increased values in 59 patients (87%). Barrier impairment was severe in 26 patients (QAlb >20) and mild to moderate in the others. Only 8 patients presented CSF lactate concentrations >3.5 mmol/l. 17 patients showed values within a range of 2.6-3.5 mmol/l, while the remaining patients presented CSF lactate concentrations <2.6 mmol/l.

**Table 2:** ᅟ

Clinical features	Patients (number)	Cells/μl	Lactate (mmol/l)	Protein (mg/l)	Albumin ratio	Intrathecal synthesis (no)			OCB (number)
						IgM	IgG	IgA	
All patients	68	164 (2–1025)	2.3 (1.2–5.3)	1034 (312–3905)	16.3 (3.7–65.9)	50/68	32/68	22/68	61/68
Cranial nerve palsy	34	164 (8–1025)	2.3 (1.3–4)	946 (312–3558)	15.8 (3.7–59.4)	27/34	17/34	9/34	30/34
Facial nerve palsy	29	165 (8–1025)	2.4 (1.3–4)	960 (312–3160)	15.8 (3.7–59.4)	24/29	16/29	8/29	25/29
Isolated facial nerve palsy	11	163 (8–1025)	1.9 (1.3–2.9)	587 (312–1800)	10.5 (3.7–27.4)	8/11	6/11	2/11	10/11
+ Radiculitis	8	117 (52–777)	2.5 (1.9–3.5)	1348 (489–2235)	21.5 (6.9–42.1)	8/8	6/8	3/8	8/8
+ Meningitis	6	408 (120–720)	2.4 (1.9–2.8)	1233 (329–1490)	17.9 (5.6–21.2)	5/6	2/6	3/6	4/6
+ Radiculitis + meningitis	4	(65, 382, 388, 767)	(1.5, 2, 3.4, 4)	(960, 1018, 1611, 3160)	(14, 15.8, 24.8, 59.4)	3/4	2/4	0/4	3/4
Oculomotor nerve palsy	5	155 (9–700)	(2.7, 1.8, 1.7, nd, nd)	703 (537–3558)	10.6 (5.9–51.2)	3/5	1/5	1/5	5/5
Isolated oculomotor nerve palsy	2	(9, 155)	(1.7, nd)	(568, 3558)	(10.6, 51.2)	1/2	0/2	0/2	2/2
+ Meningitis / radiculitis	3	(27,198, 700)	(1.8, 2.7, nd)	(537, 703, 1320)	(5.9, 9.3, 20.7)	2/3	1/3	1/3	3/3
Radiculitis	17	195 (2–800)	2.1 (1.3–4.1)	1315 (411–3600)	20 (5.2–63.8)	11/17	4/17	3/17	15/17
Isolated radiculitis	14	200 (2–703)	2.1 (1.3–3.7)	1135 (411–3600)	17.8 (5.2–62.8)	10/14	3/14	2/14	12/14
+ Meningitis	3	52, 348, 800	2, 3, 4.1	1315, 2334, 2471	21.6, 18.6, 41.8	1/3	1/3	1/3	3/3
Encephalitis	8	40 (8–494)	2.6 (1.2–4.1)	1046 (427–3905)	15.3 (8.4–65.9)	7/8	7/8	6/8	8/8
Chronic course	5	62 (8–140)	3 (1.6–4.1)	1220 (583–3905)	18.4 (8.5–65.9)	4/5	5/5	4/5	5/5
Acute / subacute onset	3	(13, 17, 494)	(1.2, 1.6, 4.1)	(427, 639, 3290)	(8.4, 11.7, 52.3)	3/3	2/3	2/3	3/3
Myelitis	5	175 (120–738)	3.4 (2.3–5.3)	1515 (977–3090)	25.9 (14.5–51.5)	3/5	3/5	3/5	5/5
Meningitis	4	(136, 211, 260, 328)	(1.8, 2.5, 2.9, 3.2)	(643, 677, 749, 1985)	(8.7, 8.8, 11.9, 27.1)	2/4	1/4	1/4	3/4

Oligoclonal bands restricted to the CSF were found in 61 patients (90%) indicating intrathecal IgG synthesis. Five of these patients showed a combination of oligoclonal bands restricted to the CSF and identical oligoclonal IgG bands in CSF and serum (type 3).

Quantitative intrathecal synthesis of immunoglobulins (Reiber-Felgenhauer graphs) of either IgM, or IgG, or IgA occurred in 55 patients (81%). Intrathecal synthesis of IgM was found in 50 patients (74%), IgG synthesis was found in 32 patients (47%), and IgA synthesis was found in 22 patients (32%). A combined three-class reaction of intrathecal synthesis of IgG, IgM, and IgA was found in 16 patients (24%). In addition, two-class reactions with the following combinations were found: IgM + IgG in 11 patients (16%), IgM + IgA in three patients (4%), and IgG + IgA in three patients (4%). Isolated IgM synthesis was found in 20 patients (29%) and isolated IgG synthesis was found in two patients (3%). Isolated IgA synthesis was absent.

When oligoclonal bands were included as a sign of intrathecal IgG synthesis (qualitative method) a combined three-class reaction of immunoglobulins was then found in 19 patients (28%). Two-class reactions with the combinations of IgM + IgG in 31 patients (46%) and IgA + IgG in 3 patients (4%) were detected. Isolated IgG synthesis was found in 9 patients (13%). A two-class reaction of IgM + IgA as well as isolated IgM and isolated IgA synthesis did not occur.

As described above, 55 patients showed quantitative intrathecal synthesis of immunoglobulins (Reiber-Felgenhauer graphs). In this group, 46 patients (84%) showed highest amounts of IgM synthesis followed by IgA in 5 patients (9%), and IgG in 4 patients (7%).

### Borrelia burgdorferi sensu lato antibodies

**Fig. 2:**
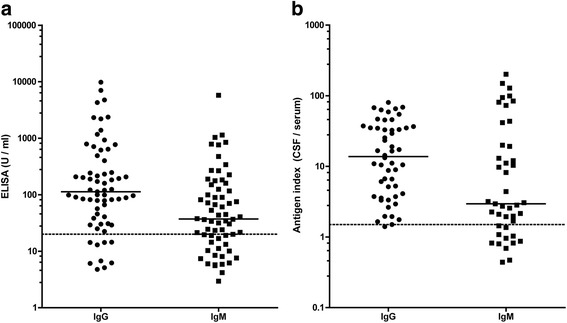
Borrelia burgdorferi sensu lato antibody synthesis in patients with neuroborreliosis. In **a** serum IgG and IgM antibody results from ELISA analyses are shown. Serum IgG and IgM values >24U/ml indicate positive results. In **b** antigen index of Borrelia specific IgG and IgM is shown which indicates specific intrathecal antibody synthesis. Antigen index values ≥ 1.5 indicate an intrathecal synthesis

Borrelia burgdorferi sensu lato specific IgG or IgM antibodies, as measured by ELISA, were tested positive in serum in 64 patients (94%; Fig. 2). In 34 patients both IgG and IgM positive antibodies were present (50%). 23 patients were positive for IgG but negative for IgM antibodies (34%). Only seven patients were positive for IgM antibodies but negative for IgG (10%).

Four patients lacked Borrelia burgdorferi sensu lato specific IgG or IgM antibodies in the serum (6%). By using immunoblot two of these patients showed negative serum results as well. The other two patients showed only one positive band for IgG (VIsE) and one positive band for IgM (p41 or OspC) in serum. However, in these four patients CSF analysis revealed pleocytosis (42–738 cells/μl), CSF oligoclonal bands (type 2), and Borrelia burgdorferi sensu lato specific IgM and IgG in the CSF. These patients suffered from either meningitis, myelitis, facial palsy, or a combination of facial palsy, meningitis, and radiculitis. Symptom duration ranged between 2 days and 3 weeks.

Figure 2 represents IgG and IgM AI values to Borrelia burgdorferi sensu lato. CSF results for both IgG and IgM were available from 52 patients, while in 16 patients either IgG or IgM were available. In the group of patients with available data for IgG and IgM, 29 showed intrathecal synthesis for both IgG and IgM, while in 20 patients intrathecal synthesis was limited to IgG and in 3 patients to IgM only.

## Discussion

**Fig. 3:**
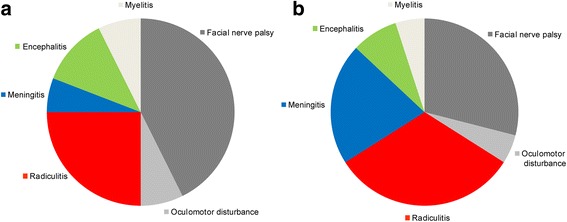
Distribution of neurological symptoms/diagnoses in patients with neuroborreliosis. In **a** the pivotal symptoms are shown that were the reason for presentation in our hospital. In fact, some patients showed more than one neurological symptom. In these patients symptoms did not occur at the same time and back pain (radiculitis) was usually the first symptom, followed by meningitis (headache), and finally by cranial nerve palsy. In **b** the distribution of all symptoms is shown. This reveals that signs of meningitis were often found in patients with neuroborreliosis, but, as indicated in A, usually did not cause admission to a hospital

In this study we classified patients with neuroborreliosis depending on neurological deficits that were the reason for presentation in a hospital. Here, we found that signs of meningitis were often found in patients with neuroborreliosis, but usually did not cause admission to a hospital (Fig. 3). In our cohort, cranial nerve palsy was the most frequent deficit which caused admission to a hospital followed by painful radiculitis, encephalitis, myelitis, and meningitis.

Patients presented either with isolated symptoms/deficits or a combination of multiple neurological symptoms. In patients with a combination of deficits, back pain (radiculitis) was the first symptom, followed by meningitis (headache), and finally by cranial nerve palsy in all but one patients. Our results are in line with a previous report showing that cranial nerve palsy is not the initial symptom when multiple neurological symptoms are present [23]. The diagnosis of cranial nerve palsy and myelitis is straightforward for neurologists and lumbar puncture is usually performed. However, diagnosis of neuroborreliosis can be difficult in other cases. Patients with radiculitis either reported back pain only or radiating pain into the limbs with or without paresis. Pain symptoms were rather unspecific and could therefore be easily misinterpreted e.g. as herniated disc. In three patients limb palsy due to radiculitis occurred suddenly, which led to initial misdiagnosis stroke.

Encephalitis seems to be another diagnostic challenge in neuroborreliosis. In our cohort, one patient presented with severely altered consciousness suggesting delirium for hours with the need of intensive care. MRI of the brain showed signs of basal vasculitis without infarction. Stroke on the basis of cerebral vasculitis is indeed a rare manifestation of neuroborreliosis in both adults and children and was predominantly described in case reports or series [4, 7, 24–43]. In these cases typical stroke-like symptoms such as paresis, dysarthria, and/or aphasia have been reported. Only in few cases paresis combined with delirium were described. Interestingly, our patient presented with acute delirium due to vasculitis without stroke. Since such patients are frequently hospitalized in psychiatric institutions, psychiatrists should be aware of possible neuroborreliosis in patients with acute delirium. Another five patients in our cohort showed symptom duration of more than 6 months and were classified as chronic encephalitis. These patients were older than 65 years and suffered from cognitive impairment and gait disorder suspicious for normal pressure hydrocephalus (NPH). However, lumbar puncture with drainage of CSF did not change their symptoms. Instead, CSF analysis revealed pleocytosis and intrathecal synthesis of Borrelia specific antibodies indicating neuroborreliosis. Improvement of clinical symptoms after antibiotic treatment occurred in all of these cases. Similar cases of neuroborreliosis with symptoms mimicking NPH were already described in some case reports [44–48]. Pseudotumor cerebri presents another disease which might be related to neuroborreliosis. In several reports typical symptoms of pseudotumor cerebri such as headache and papilledema and an elevated CSF opening pressure have been described predominantly affecting children [49–60]. In these patients antibiotic treatment led to normalization of symptoms and CSF pressure.

The seasonal distribution of symptom onset might be of some help in suspicious cases. The majority of our patients (60%) presented between July and September. Similar results were communicated in earlier studies in Danish, Swedish, and Finish populations [6, 23, 61, 62]. Nevertheless, in our cohort neuroborreliosis was diagnosed in every month of the year except February. Therefore, low-risk months are unreliable for ruling out neuroborreliosis as already suggested [23].

For detection of Borrelia burgdorferi sensu lato infection serum is usually analyzed using ELISA with subsequent confirmation by immunoblot. Remarkably, four of our patients with neuroborreliosis lacked positive results for Borrelia infection in the blood (6%) but additional analysis of the CSF revealed pleocytosis and intrathecal production of antibodies against Borrelia burgdorferi sensu lato. Thus, negative blood results do not necessarily rule out neuroborreliosis. The same conclusion was drawn in previous studies [63–65]. Henningsson and collegues found Borrelia specific antibodies in the CSF but not in serum in 25 of 150 patients (17%) by using ELISA at the time of diagnosis [64].

Our CSF data are largely in line with previous observations [10, 64, 66, 67]. We found elevated CSF cell counts in all but one patient. Blood-CSF barrier dysfunction occurred in 87% patients. Oligoclonal bands restricted to the CSF were found in 90% patients indicating intrathecal IgG synthesis. Quantitatively elevated intrathecal synthesis of immunoglobulins (either IgM, or IgG, or IgA) based on the calculation method of Reiber-Felgenhauer prevailed in 81% of the patients. Intrathecal synthesis of IgM was found in 74%, IgG synthesis was found in 47%, and IgA in 32% of the patients. Interestingly, isolated intrathecal synthesis of IgM or IgA was not found and was accompanied by positive oligoclonal bands in all cases. These results demonstrate that neuroborreliosis is accompanied by pronounced immunological CSF changes.

Active neuroborreliosis without pleocytosis is controversially discussed and three scenarios are suggested [3, 64, 66, 68]. The most common interpretation is that the majority of cases with intrathecal Borrelia specific antibody production without CSF pleocytosis are due to a previous neuroborreliosis. However, other authors attribute the absence of pleocytosis in some cases to a prolonged disease. Henningsson and colleagues described four cases with normal CSF cell count and a long history of illness (from 36 weeks till 6 years) [64]. Remarkably these four patients displayed the highest Borrelia specific IgG indices in their cohort and they have speculated that the initial pleocytosis might have been already resolved by the time of lumbar puncture probably reflecting the natural course of the disease. Conversely, others suggested that the lack of elevated CSF cell count may be associated with the brevity in disease duration in some cases. Rupprecht and colleagues described two cases with normal cell counts who were successfully treated with antibiotics [68]. Repeated CSF analyses after 6 or 14 days indeed discovered an increase in cell counts and an elevation of the Borrelia specific antibody index confirming active neuroborreliosis. Our cohort included only one patient without pleocytosis whose symptoms resolved completely after treatment with ceftriaxone. However, this patient did not undergo a second lumbar puncture, and thus, according to the diagnostic criteria there is a limitation regarding a definitive diagnosis. In our opinion, antibiotic treatment in such cases is justified with the important restriction that such a therapy has not been conducted before. The chemokine CXCL13 has been evaluated as a possible CSF biomarker for detection of acute neuroborreliosis [69–71]. CXCL13 analysis might help in such cases to differentiate between acute neuroborreliosis and previous infection. However, CXCL13 is not yet in use as a routine parameter due to concerns about the specificity [68].

## Conclusions

This study shows that most, but not all patients with neuroborreliosis presented with typical neurological findings and consistent CSF changes. Uncommon presentations such as acute delirium and stroke-like symptoms underline the variety of symptoms. A thorough CSF analysis is considered essential for a reliable diagnosis of neuroborreliosis.

## Abbreviations

CNS: Central nervous system
CSF: Cerebrospinal fluid
MRI: Magnetic resonance imaging
OCB: Oligoclonal bands
QAlb: CSF-serum albumin quotient
